# The social capital effect on HIV/AIDS preventive efforts: a meta-analysis

**DOI:** 10.25122/jml-2021-0348

**Published:** 2022-10

**Authors:** Reny Nugraheni, Bhisma Murti, Muhammad Eko Irawanto, Endang Sutisna Sulaiman, Eti Poncorini Pamungkasari

**Affiliations:** 1 Doctoral Program of Public Health, Universitas Sebelas Maret, Surakarta, Indonesia; 2 Faculty of Health Technology and Management, Institut Ilmu Kesehatan Bhakti Wiyata, Kediri, Indonesia; 3 Skin and Sexual Health Sciences, Universitas Sebelas Maret, Surakarta, Indonesia; 4 Medical Sciences, Universitas Sebelas Maret, Surakarta, Indonesia

**Keywords:** social capital, HIV/AIDS, prevention, condom use

## Abstract

HIV/AIDS is one of the sexually transmitted diseases that cause death worldwide. Its prevalence increases due to low prevention behaviour. The study aimed to estimate the effect of social capital on HIV/AIDS preventive efforts. This study was a meta-analysis and systematic analysis. We retrieved articles from PubMed, Science Direct, and Google Scholar from 2008–2021. The inclusion criteria were full-text articles with observational design and articles published in English. We focused on the problems of the PICO study, namely: population=men and women who were sexually active; intervention=high social capital; comparison=low social capital; outcome=HIV/AIDS prevention efforts. The articles were collected using the PRISMA flow diagram. Meta-analysis was performed using RevMan 5.3 with a random effect model. The study included 12 articles. The likelihood of sexually active men and women with high social capital to perform HIV/AIDS prevention efforts was 1.55 times higher than those with low social capital (aOR=1.55; CI 95%=1.11 to 2.16; p=0.009).

## INTRODUCTION

HIV/AIDS is one of the primary sexually transmitted diseases worldwide. The human immunodeficiency virus (HIV) targets the immune system. It weakens people's defenses against many infections and some cancers that people with healthy immune systems can fight [1]. As the virus destroys and disrupts the function of immune cells, infected people gradually become immunocompromised. The most advanced stage of HIV is acquired immune deficiency syndrome (AIDS), which can take years to develop, depending on the individual. AIDS is defined by the development of certain cancers, infections, or other long-term severe clinical manifestations [2].

HIV/AIDS is a significant public health problem worldwide, with 34.7 deaths per year. There is no cure for HIV infection, but it can be controlled by increasing access to effective HIV prevention, diagnosis, treatment and care. At the end of 2020, an estimated 37.6 million people were infected with HIV, more than two-thirds in the WHO African Region. Six hundred ninety thousand people die from HIV-related causes, and 1.5 million people become infected with HIV.

There are many feasible efforts to prevent HIV/AIDS, including using a condom during sexual intercourse, being monogamous, getting tests and counselling for HIV and STIs, taking antiretroviral (ARV) drugs for prevention, reducing syringe abuse among injected drug users, and abolishing mother-to-child transmission. Some of the prevention efforts are affected by social capital, which represents the norms and networks that allow people to collaborate [3]. Social capital is identified in several theoretical models as a potential determinant and is measured with several indicators such as trust, reciprocity, sense of belonging, network bonding, and social participation. Social capital has beneficial impacts related to the prevention of HIV/AIDS incidences [4].

Several studies have been conducted to discover the effect of social capital toward HIV/AIDS prevention efforts. One study [5] revealed that social capital affects the risk of HIV/AIDS transmission [6]. Another study [7] states that an individual's social money depends on social relationships [8]. Social capital in a particular group can affect the protection against HIV. This is supported by another study [9] that mentions that public health strategies that attempt to provide HIV prevention, treatment, and care will benefit from recognizing the social capital that exists in the community. Social capital as a resilience strategy is utilized to access healthcare services by raising solidarity and social support to ensure the reception and sustainability of HIV prevention and promotion efforts.

The increased HIV/AIDS incidence caused by the low social capital-related prevention efforts, supported by several studies, encouraged the researcher to incorporate and analyze the current literature and draw a conclusion from the studies that discuss the effect of social capital on HIV/AIDS prevention efforts.

## MATERIAL AND METHODS

### Study Design

The study was a systematic review and meta-analysis study. Articles used in the study were obtained from several databases: PubMed, Science Direct, and Google Scholar. Keywords to find the articles were the following: "social capital" AND "HIV/AIDs" OR "social capital" AND "prevention HIV/AIDs" AND "condom use".

### Inclusion Criteria

Articles included in the study were full-text articles with a cross-sectional study design and excellent social capital study interventions with outcomes in the form of HIV/AIDS prevention. The articles used in the study were published in English.

### Exclusion Criteria

Articles excluded from the study were random controlled trials, case-control studies, quasi-experiments, protocols, and pilot studies. Other exclusion criteria were: articles not published in English, studies not reporting aOR, studies with overestimated effects, or with a CI too broad.

### Operational Definition

Articles search was conducted by considering the appropriateness criteria, which was defined by using the PICO model. In the study, the population was men and women who were sexually active with the intervention in the form of high social capital. The comparison was low social capital, and the outcome was HIV/AIDS preventive efforts.

Social capital was an approach that could affect individual and community health. The instruments used were questionnaires with the categorical scale of measurement.

HIV/AIDS preventive efforts were the activities to prevent sexually transmitted diseases. The instruments used were questionnaires with categorical scales of measurement.

### Data Analysis

Data processing was conducted using Review Manager (RevMan 5.3) by calculating effect size and heterogeneity to determine the incorporated models of the study and formulate the final result of the meta-analysis.

## RESULTS

**Figure 1:**
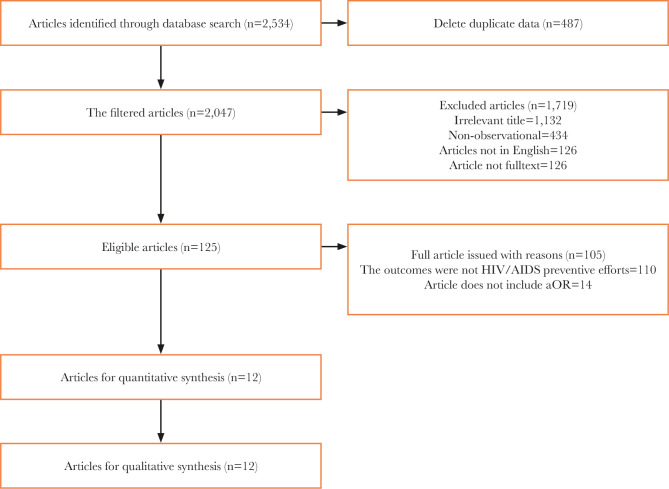
PRISMA flow diagram.

**Figure 2:**
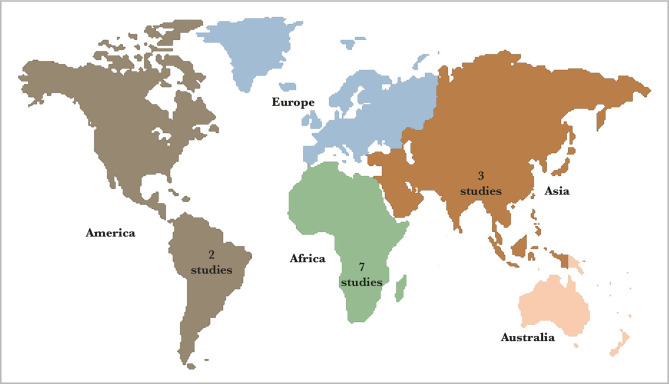
Research Map for the Effect of Social Capital on HIV/AIDS Prevention Efforts.

The process of article search in journal databases can be viewed in Figure 1. Figure 2 indicates the areas the articles were taken from, following inclusion criteria. The reports were obtained from 3 continents, Asia, Africa, and America (Table 1).

**Table 1:** Research Quality Assessment of the Effect of Social Capital on HIV/AIDS Preventive Effort.

Primary Study	Criteria												Total
	1	2	3	4	5	6	7	8	9	10	11	12	
**[9]**	1	1	1	1	1	1	1	1	1	1	1	1	12
**[10]**	1	1	1	1	1	0	1	1	1	1	1	1	11
**[11]**	1	1	1	1	1	1	1	1	1	0	1	1	10
**[12]**	1	1	1	1	1	1	1	1	1	1	1	1	12
**[13]**	1	1	1	1	1	1	1	1	1	0	1	1	11
**[14]**	1	1	1	1	1	1	1	1	1	1	1	1	12
**[15]**	1	1	1	1	1	1	1	1	1	0	1	1	10
**[16]**	1	1	1	1	1	0	1	1	1	1	0	1	12
**[17]**	1	1	1	1	1	1	1	1	1	1	1	1	12
**[18]**	1	1	1	1	1	0	1	1	1	1	1	1	11
**[19]**	1	1	1	1	1	1	1	1	1	0	1	1	11
**[20]**	1	1	1	1	1	1	1	1	1	1	1	1	12

Answer 1 – yes; answer 0 – no.

The Study Quality Assessment used Critical Appraisal CEBMa (Center for Evidence-Based Medicine), which consisted of 12 questions as the following:

1. Does the aim discuss the focus/problem of the study?
2. Is the study method (study design) appropriate to answer the research questions?
3. Is the sampling method clearly stated?
4. Does the sampling method potentially bring out bias (of selection)?
5. Does the selected sample represent the intended population?
6. Is the sample order based on pre-study consideration?
7. Is the satisfying response achieved?
8. Is the study instrument valid and reliable?
9. Is the statistical significance measured?
10. Is a confidence interval given for the main result?
11. Is there any unmeasured confounding factor?
12. Is it feasible to apply the impact to your study?

**Figure 3:**
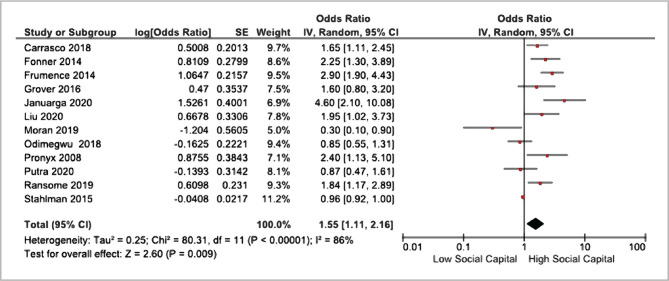
Forest plot for the meta-analysis.

Based on the analysis of Table 2 and Figure 3, twelve articles highlight the potential of sexually active men and women with high social capital. HIV/AIDS prevention efforts were 1.55 times higher than those with low social capital (AOR=1.55); CI 95%=1.11 to 2.16; p=0.009). The meta-analysis had a high heterogeneity effect (I^2^=86%; p=0.009), so the inclusion was conducted using the random effect model method.

**Table 2:** Description of primary studies included in the meta-analysis.

Studies	Countries	Study Design	Sample	P (Population)	I (Intervention)	C (Comparison)	O (Outcome)
[6]	Swaziland	Cross-sectional	326	Sex workers	Social cohesion value of condom users was high*, the value of social cohesion afraid to seek healthcare service was high, the value of social cohesion participating in meetings about prostitutes' right promotions was high	Social cohesion value of not using a condom was high*, the value of social cohesion afraid to seek for healthcare service was low, the value of social cohesion participating in meetings about prostitutes' rights promotions was low	HIV/AIDS preventive efforts
[11]	China	Cross-sectional	327	VCT users (MSM and prostitutes)	Social capital mainly shared vision was high*, social capital particularly network was high, social capital particularly support was high	Social capital mainly shared vision was low*, social capital particularly network was down, social capital particularly support was low	HIV/AIDS preventive efforts
[12]	Indonesia	Cross-sectional	200	Sex workers	Social capital peer support was high; social capital trust was high*, social capital social participation was high	Social capital peer support was low; social capital trust was low*, and social capital social participation was low	HIV/AIDS preventive efforts
[14]	Ivory Coast	Cross-sectional	1,301	Man Sex Man (MSM)	A score of social cohesion was high*, score of social cohesion was low	A score of social cohesion was low*	HIV/AIDS preventive efforts
[16]	South Africa	Cross-sectional	375	Sexually active men	Cognitive, the social capital of using a condom was high*, the cognitive, social capital of knowledge was higher, social capital cognitive of communication was good	Cognitive, the social capital of not using a condom was low*, social capital cognitive of knowledge was inadequate, the cognitive, social capital of communication was inadequate	HIV/AIDS preventive efforts
[17]	Lesotho	Cross-sectional	530	Man Sex Man (MSM)	A score of social cohesion was high*	A score of social cohesion was low*	HIV/AIDS preventive efforts
[18]	Dominican Republic	Cross-sectional	228	Sex workers	A score of social cohesion was high*	A score of social cohesion was low*	HIV/AIDS preventive efforts
[19]	Tanzania	Cross-sectional	3,586	Sexually active men and women	Individual structural social capital was moderate*; individual cognitive, social capital was moderate, privileged	Individual structural social capital was low*; individual cognitive, social capital was low, underprivileged	HIV/AIDS preventive efforts
[20]	Indonesia	Cross-sectional	406	Sex workers	Relatively were not assured that their peers were committed to using condoms*, instead felt safe at work, rather were respected by people in the area	Were not assured that their peers were committed to using condom*, felt uncomfortable at work, and were not respected by people in the area	HIV/AIDS preventive efforts
[21]	Swaziland	Cross-sectional	326	Man Sex Man (MSM)	High vs. Low social cohesion*, High vs. Low cohesion	Moderate vs. Low social cohesion*	HIV/AIDS preventive efforts
[22]	South Africa	Cross-sectional	1,477	Sexually active men and women	High social capital, moderate social capital	Low social capital*	HIV/AIDS preventive efforts
[23]	USA	Cross-sectional	1,445	Sexually active men and women	High social capital*	Low social capital*	HIV/AIDS preventive efforts

* – variables included in the meta-analysis study.

**Figure 4:**
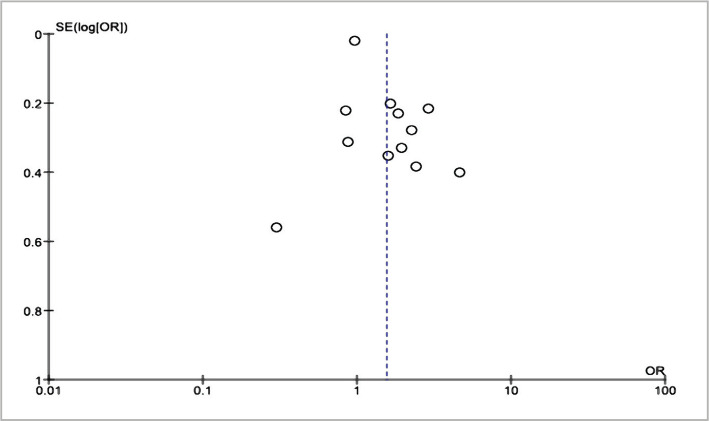
Funnel plot for the meta-analysis of the social capital effect on HIV/AIDS preventive efforts.

There was a publication bias which was marked by the symmetrical plots between the left and the right (Figure 4). There were five plots on the right, four plots on the left, and three plots precisely on the vertical line.

## DISCUSSION

The studies on the social capital effect on HIV/AIDS preventive efforts consisted of 12 primary studies spread across three continents Asia, Africa, and America. Among others are Indonesia, China, Swaziland, Ivory Coast, Lesotho, Afrika Selatan and the USA.

The systematic review and meta-analysis results were presented as forest plots and funnel plots. The forest plot indicates brief information about each study included in the meta-analysis and estimates the results. The forest plot shows the size of variation (heterogeneity) among the study results [8].

### Social Capital Effect on HIV/AIDS Preventive Efforts

The meta-analysis results of the 12 articles regarding the effect of social capital on HIV/AIDS preventive efforts were summarized in the forest plot. The forest plot in Figure 4 indicates that the likelihood of sexually active men and women with high social capital performing preventive efforts was 1.55 times higher than those with low social capital (aOR=1.55; CI 95%=1.11 to 2.16; p=0.009). A study supports the correlation between social capital, risky sexual behaviours, and HIV/AIDs. One study identified that the social cohesion approach is different between men and women [24]. Women have a lower risk of HIV/AIDS than men. This is supported by a woman's self-efficacy in persuading her partner to use protection for safe sexual intercourse to reduce the risk or prevent HIV/AIDS [23]. This corresponds with a study which identified that social capital affects HIV/AIDS preventive efforts. The study used social cohesion for social capital measurement, divided into several variables: peer support, trust, and social participation, which were then divided into high and low levels. The study discovered that female social workers with a high score of social cohesion trust are more likely to perform HIV/AIDS prevention efforts by using a condom during sexual intercourse [25].

Another study regarding social cohesion and HIV/AIDS-related behaviour and risk factors identified that sex workers with high social cohesion scores also perform HIV/AIDS preventive efforts. High social participation is also related to increased HIV/AIDS preventive efforts. The HIV/AIDS efforts performed in the study were the consistent use of a condom by all partners of sex workers.

This is supported by a study which mentions that social capital significantly affects HIV/AIDS risk prevention. The study divides the social capital assessment into cognitive and structural social capital. At the mental and social capital level, most men perform HIV/AIDS prevention compared to those at the structural social capital level. Men are more open in discussing sex and reporting condom use more effectively in HIV/AIDS prevention [25].

This study has several drawbacks, including search bias. Because the researcher uses only three databases, namely PubMed, Google Scholar and ScienceDirect, it ignores other sources. Another limitation is the language bias since the study only uses articles published in English and ignores articles in other languages.

## CONCLUSION

Based on the result of the 12 primary studies, it can be concluded that the likelihood of sexually active men and women with high social capital performing HIV/AIDS preventive efforts is 1.55 times higher than that of those with low social capital.

## Personal thanks

The authors thank Prof. Ari Natalia Probandari, dr., MPH, PhD from Universitas Sebelas Maret, Surakarta, Indonesia, for her helpful advice on this study.
